# Pulmonary Sclerosing Hemangioma Presenting As Perihilar Lung Nodule in a Patient With Papillary Thyroid Carcinoma: A Case Report and Review of the Literature

**DOI:** 10.7759/cureus.104146

**Published:** 2026-02-23

**Authors:** Hunter Stecko, Marissa Guo, Aaron Guo, William MacDonald, Desmond D'Souza

**Affiliations:** 1 Thoracic Surgery, The Ohio State University Wexner Medical Center, Columbus, USA; 2 Pathology, The Ohio State University Wexner Medical Center, Columbus, USA

**Keywords:** adult thoracic surgery, difficult diagnosis, histopathology and immunohistochemistry, incidental lung nodule, pulmonary sclerosing pneumocytoma, rare tumors

## Abstract

Pulmonary sclerosing pneumocytoma is a rare, typically asymptomatic, tumor with potential for malignant transformation. Diagnosis is frequently difficult due to its rarity and proclivity to mimic other pathologies. Here, we describe a particularly challenging case of a patient with a personal and familial history of other malignancies who presented with an incidental lung nodule, found to be pulmonary sclerosing pneumocytoma on pathologic examination after an anatomic lung resection. We conclude with a review of the natural history, clinical and histopathological presentation, and management options for this rare entity.

## Introduction

Pulmonary sclerosing pneumocytoma (PSP), previously called pulmonary sclerosing hemangioma, is a rare benign tumor with uncertain histogenesis, although studies have suggested that these lesions arise from primitive respiratory epithelium [1]. This proposed epithelial origin is further supported by the consistent expression of thyroid transcription factor-1 (TTF-1) and epithelial membrane antigen (EMA) in both surface and round cell populations, as well as recurrent activating AKT1 mutations, implicating dysregulation of the PI3K/AKT/mTOR pathway in PSP. PSP has a strong female predominance and is commonly diagnosed in the fifth decade [2]. While there have been reports of PSPs causing symptoms, such as cough, hemoptysis, chest pain, and fever, the majority of patients are asymptomatic, and the discovery of PSP is often incidental. On computed tomography (CT) scan, PSP generally appears as a singular ovoid lesion with smooth borders. While PSP has a propensity to be located peripherally, other presentations, including endobronchial involvement, have been described [3].

Pathologically, PSP comprised two cell types: cuboidal cells resembling bronchiolar epithelium and type II alveolar pneumocytes lining the surfaces of papillary and tubular structures, and interstitial round cells with relatively consistent morphology [1,4]. Immunohistochemistry typically shows positive staining for TTF-1 and EMA [1,4]. Alterations to mTOR-related pathway genes, particularly AKT1, are frequently implicated in the pathogenesis of PSP [5], though mutations in BRAF and IDH1 have also been reported [6,7].

The clinical presentation of PSPs has been found to mimic carcinoid tumors [8]. Carcinoid tumors are frequently diagnosed with nuclear imaging using somatostatin receptor (SSTR)-targeted radiotracers, such as (86)Ga-DOTATATE and (86)Ga-DOTANOC [9]. However, PSP may also show avidity on these scans, possibly due to the expression of SSTRs by respiratory epithelial tissues [10]. The rate at which this occurs is currently unreported in the literature due to the rare nature of PSP. To add further complexity, some lesions have been demonstrated to have concurrent carcinoid tumors with PSP [11]. Additional case descriptions are needed to inform clinicians on how to approach the diagnosis and management of this rare tumor. Differentiating PSP from carcinoid tumor is particularly important given the difference in need for surveillance and approach to resection in patients with PSP vs. carcinoid tumor. For this reason, we herein present the case of a patient with diagnostically complex PSP.

## Case presentation

**Figure 1:**
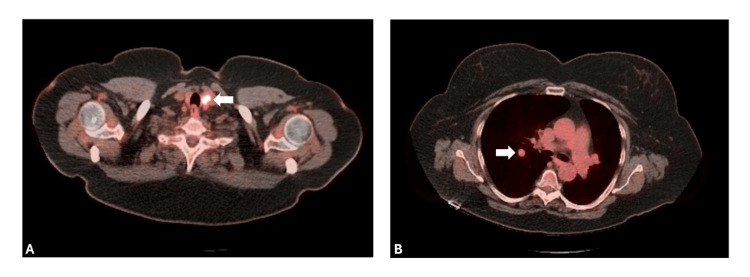
Positron emission tomography/computed tomography imaging of the neck and chest (A) A 1.2 cm left thyroid mass demonstrating high 18F-fluorodeoxyglucose (FDG) avidity (SUV 9) (white arrow). (B) Incidental finding on the same diagnostic scan demonstrating a 1.2 cm right perihilar lung nodule with mild FDG avidity (SUV 3.5) (white arrow).

A 65-year-old woman with a remote smoking history presented with an incidentally identified lung mass while undergoing work-up for a thyroid nodule. Her medical history otherwise included hypertension, hyperlipidemia, type 2 diabetes, and obstructive sleep apnea. Her family history was significant for multiple instances of breast cancer among female relatives, ovarian cancer, and thyroid cancer. Positron emission tomography (PET)/CT scan demonstrated 18F-fluorodeoxyglucose (FDG) uptake by the thyroid lesion and adjacent lymph nodes, suggestive of malignancy (Figure 1A). In addition, it showed a well-circumscribed right-sided perihilar lung nodule with a standardized uptake value (SUV) of 3.5 (Figure 1B). She underwent total thyroidectomy with left neck dissection, and the final pathological diagnosis was consistent with locally advanced papillary thyroid carcinoma with lymph node metastases. Notably, post-procedural radioactive iodine uptake studies showed no uptake in the previously identified lung mass.

**Figure 2:**
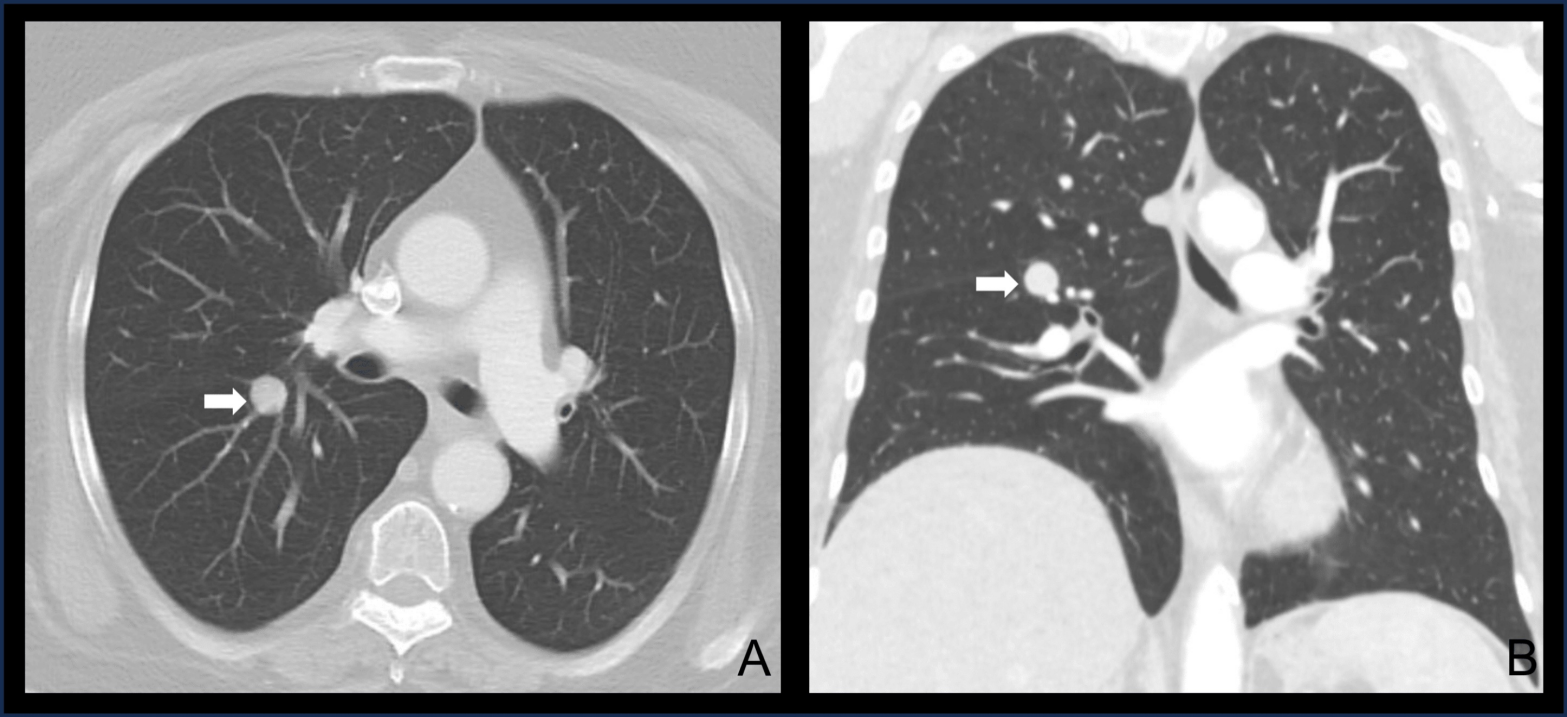
Pre-operative computed tomography scan of the chest A computed tomography scan demonstrating an axial (A) and coronal (B) view of a round, perihilar mass located within the patient’s right lower lobe (white arrow).

Following her recovery, she was referred to thoracic surgery for further evaluation. A CT chest scan demonstrated a round, noncalcified nodule measuring 1.6 cm by 1.5 cm in the right lower lobe (RLL) (Figure 2). Endobronchial biopsy of the RLL nodule and mediastinal lymph nodes via navigational bronchoscopy was non-diagnostic. Subsequent CT-guided percutaneous biopsy of the pulmonary lesion revealed atypical cells; however, the yield was insufficient for a conclusive diagnosis. A SSTR-targeted PET/CT scan was also obtained to evaluate for a neuroendocrine tumor, which demonstrated a stable RLL nodule with mild tracer avidity. Pulmonary function testing was within normal limits.

Ultimately, the patient was recommended for robotic-assisted right lower lobectomy given concern for carcinoid tumor. Dissection of thoracic lymph nodes from stations 2, 4, 7, 9, 10, and 11 was performed during the operation. The patient tolerated the procedure well and had an uncomplicated post-operative course. Given her extensive family history of cancer, she eventually underwent somatic genomic testing, which revealed no pathogenic variants, including any within MLH1, MSH2, MSH6, PMS2, EPCAM, BRCA1, or BRCA2. 

Her inpatient course was uncomplicated, and she was discharged on post-operative day 2 in good condition. She was readmitted to an outside hospital on post-operative day 5 due to shortness of breath. Residual hydropneumothorax was visualized on a spiral CT scan obtained to rule out pulmonary embolism as a cause of her dyspnea. She was discharged on home oxygen, which she discontinued within a week following her discharge on post-operative day 6. 

**Figure 3:**
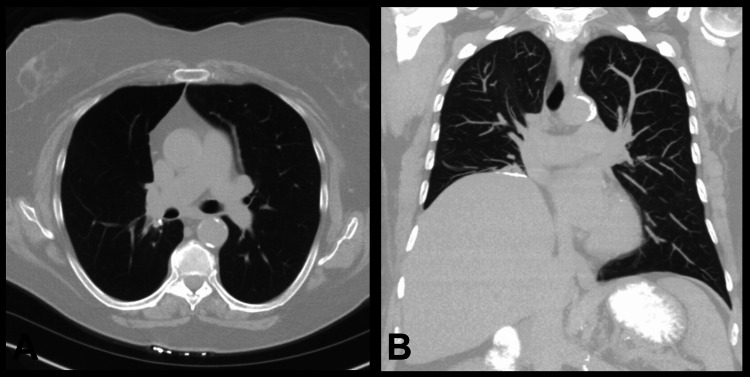
Six-month post-operative computed tomography scan of the chest A computed tomography scan demonstrating an axial (A) and coronal (B) view of the patient’s lungs following resection of the right lower lobe. Normal post-operative changes are demonstrated and no recurrence of the tumor is visualized.

On one-month follow-up, she was recovering well, and her surgical incisions were well-healed. She likewise was doing well at the six-month follow-up. Her six-month follow-up CT scan demonstrated normal post-operative changes without disease recurrence or lymphadenopathy (Figure 3).

Histopathological examination

**Figure 4:**
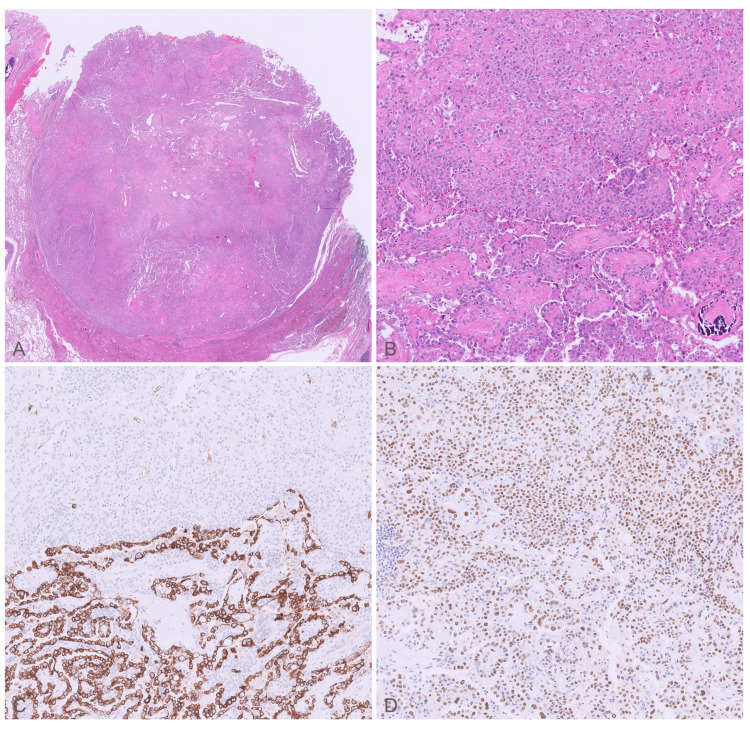
Pathologic features of the surgical specimen (A) Light microscopic examination showed a well-circumscribed tumor with variable cellularity and collagen deposition, H&E, original magnification ×2. (B) The tumor comprised two distinct cell populations, including round cells arranged in sheets (top of the panel) and surface cells lining papillary structures (bottom of the panel); original magnification ×20. The surface cells are immunoreactive with (C) cytokeratin 7 (CK7) and (D) thyroid transcription factor-1 (TTF-1), while the round cells are immunoreactive only with TTF-1; immunohistochemistry, original magnification ×20.

Gross examination showed a 1.7 cm ovoid, bulging mass with tan-white cut surfaces near the superior hilum of the RLL. No necrotic or hemorrhagic foci were present. Histopathologically, the tumor was well-circumscribed with papillary and solid architectural patterns. The surfaces of the papillae are lined by bland cuboidal cells with minimal atypia. The underlying stroma has variable collagen deposition and cellularity composed of uniform, large, round cells with distinct cell borders and abundant cytoplasm. The solid pattern consists of round cells arranged in sheets. Immunohistochemistry demonstrated diffuse TTF-1 and EMA expression in both the surface epithelial and stromal round cell populations, whereas cytokeratin expression (AE1/AE3, CK7, and CAM5.2) was restricted to the surface cells, a biphasic immunophenotypic pattern that helps distinguish PSP from primary pulmonary adenocarcinoma (Figure 4). All seven lymph nodes sampled were benign. Genomic analysis identified a 13-amino acid AKT1 internal tandem duplication, further supporting the diagnosis of PSP. Immunostains were negative for thyroglobulin, PAX8, CK5/6, p40, CK20, and no genetic mutations were detected in ALK, BRAF, EGFR, ERBB2, KEAP1, KRAS, MAP2K1, MET, NFE2L2, or PIK3CA.

## Discussion

Most PSPs appear as solitary, incidental pulmonary nodules with nonspecific findings [1], engendering a wide differential diagnosis. Here, we presented a case in which a concomitant diagnosis of thyroid cancer added further complexity to the evaluation of a patient’s pulmonary mass, highlighting the challenge of diagnosing PSPs prior to surgical resection. A previous study investigating the diagnostic accuracy of CT imaging for PSP found that up to 21% of cases were interpreted as primary lung cancer, while 16% were interpreted as a metastasis in patients with an underlying malignancy [12]. While the majority of PSPs appear hypometabolic (SUV <2.5) on FDG-PET [12], at least one other report has described a case of bilateral PSPs with high FDG uptake [13]. In our case, the primary consideration was a low-grade malignancy, such as a carcinoid tumor, given the lesion’s mild avidity on both FDG-PET and SSTR-PET.

Due to its limitations, tissue diagnosis of PSP obtained from small biopsies may lead to overdiagnosis as adenocarcinoma. PSPs are characterized by a biphasic cell population (surface and round cells) organized into four typical architectural patterns: papillary, sclerotic, solid, and hemorrhagic [1]. As a result, the reliability of specimens obtained by fine needle aspiration, core needle biopsy, or intraoperative excision for frozen section is relatively low due to the tumor’s histologic heterogeneity. PSP also shows overlap in immunoprofile with pulmonary adenocarcinoma, both of which express TTF1. Therefore, the use of wider immunostain panels, with particular attention given to their expression in tumor cell subpopulations, is preferred. However, this may or may not be possible in a setting of cytology or small biopsy samples. Nevertheless, preoperative differentiation of PSPs, the majority of which are benign and slow growing, from more aggressive neoplasms can be critical in determining the best therapeutic option for the management of these lesions.

Overall, given their rarity, a standardized approach to treating PSPs has not been established. According to the World Health Organization’s classification of lung tumors, PSPs are categorized as benign adenomas [14], and some authors have advocated for a conservative strategy of observation [15]. A retrospective study by Kim et al. found that all-cause mortality did not differ between patients with PSP and those without, irrespective of whether PSP patients underwent surgery [16]. On the other hand, there have been several instances reported of PSPs with lymph node or extrapulmonary metastases [17], suggesting that a subset of these lesions may have malignant potential. Other studies have described PSPs with rapid or unchecked growth [18], as well as malignant transformation [19]. Moreover, combined lesions have been found comprising both PSP and carcinoid tumor [11]. Such presentations provide support for early surgical resection of PSPs. This may be accomplished via local resection, such as wedge resection and tumor enucleation, segmentectomy, or lobectomy. For patients who are unable to tolerate an operation, stereotactic body radiotherapy or radiofrequency ablation represent non-surgical options [17]. In the case presented above, the decision was made to proceed with right lower lobectomy given the uncertain diagnosis and central location of the pulmonary nodule. It is also worth noting the possibility that local resection may carry a higher risk of recurrence [20]. Thus, though lung-sparing resection (i.e., wedge resection or segmentectomy) could be considered, and perhaps would be in a more well-defined disease process, such as non-small cell lung carcinoma, the lack of evidence supporting sublobar resection in PSP caused us to opt for a more extensive resection to minimize recurrence risk.

## Conclusions

PSP presents a challenging diagnostic dilemma due to its nonspecific radiological findings and histological heterogeneity. Difficulty differentiating PSP from malignant lesions, particularly in patients with a history of cancer, can lead to ambiguity regarding surgical management. PSP should remain in the differential diagnosis of well-circumscribed perihilar nodules with mild FDG avidity on PET imaging in patients with known malignancy, and definitive classification may require surgical resection in cases with inconclusive biopsy results. Additional case descriptions are needed to guide future management of these rare tumors.
